# Genomic Characterization and Clinical Outcomes of Patients with Peritoneal Metastases from the AACR GENIE Biopharma Collaborative Colorectal Cancer Registry

**DOI:** 10.1158/2767-9764.CRC-23-0409

**Published:** 2024-02-20

**Authors:** Enrique Sanz-Garcia, Samantha Brown, Jessica A. Lavery, Jessica Weiss, Hannah E. Fuchs, Ashley Newcomb, Asha Postle, Jeremy L. Warner, Michele L. LeNoue-Newton, Shawn M. Sweeney, Shirin Pillai, Celeste Yu, Chelsea Nichols, Brooke Mastrogiacomo, Ritika Kundra, Nikolaus Schultz, Kenneth L. Kehl, Gregory J. Riely, Deborah Schrag, Anand Govindarajan, Katherine S. Panageas, Philippe L. Bedard

**Affiliations:** 1 Division of Medical Oncology and Hematology, Princess Margaret Cancer Centre – University Health Network, Department of Medicine, University of Toronto, Ontario, Canada.; 2 Memorial Sloan Kettering Cancer Center, New York, New York.; 3 Dana-Farber Cancer Institute, Boston, Massachusetts.; 4 Vanderbilt-Ingram Cancer Center, Nashville, Tennessee.; 5 American Association of Cancer Research, Philadelphia, Pennsylvania.; 6 Sinai Health System, Toronto, Ontario, Canada.; 7 Department of Surgery, University of Toronto, Ontario, Canada.

## Abstract

Peritoneal metastases (PM) are common in metastatic colorectal cancer (mCRC). We aimed to characterize patients with mCRC and PM from a clinical and molecular perspective using the American Association of Cancer Research Genomics Evidence Neoplasia Information Exchange (GENIE) Biopharma Collaborative (BPC) registry. Patients’ tumor samples underwent targeted next-generation sequencing. Clinical characteristics and treatment outcomes were collected retrospectively. Overall survival (OS) from advanced disease and progression-free survival (PFS) from start of cancer-directed drug regimen were estimated and adjusted for the left truncation bias. A total of 1,281 patients were analyzed, 244 (19%) had PM at time of advanced disease. PM were associated with female sex [OR: 1.67; 95% confidence interval (CI): 1.11–2.54; *P* = 0.014] and higher histologic grade (OR: 1.72; 95% CI: 1.08–2.71; *P* = 0.022), while rectal primary tumors were less frequent in patients with PM (OR: 0.51; 95% CI: 0.29–0.88; *P* < 0.001). *APC* occurred less frequently in patients with PM (*N* = 151, 64% vs. *N* = 788, 79%) while *MED12* alterations occurred more frequently in patients with PM (*N* = 20, 10% vs. *N* = 32, 4%); differences in *MED12* were not significant when restricting to oncogenic and likely oncogenic variants according to OncoKB. Patients with PM had worse OS (HR: 1.45; 95% CI: 1.16–1.81) after adjustment for independently significant clinical and genomic predictors. PFS from initiation of first-line treatment did not differ by presence of PM. In conclusion, PM were more frequent in females and right-sided primary tumors. Differences in frequencies of *MED12* and *APC* alterations were identified between patients with and without PM. PM were associated with shorter OS but not with PFS from first-line treatment.

**Significance:**

Utilizing the GENIE BPC registry, this study found that PM in patients with colorectal cancer occur more frequently in females and right-sided primary tumors and are associated with worse OS. In addition, we found a lower frequency of *APC* alterations and a higher frequency in *MED12* alterations in patients with PM.

## Introduction

Metastatic colorectal cancer (mCRC) is the third most frequent cause of cancer death in North America (1). Peritoneal metastases (PM) are a common site of metastatic spread, occurring in approximately 13% of patients with mCRC (2). However, the incidence of PM may be underestimated because of limitations of radiological imaging, as autopsy studies indicate that up to 80% of patients who die from mCRC have PM (3). Palliative systemic chemotherapy is widely used to improve survival (4). Patients with PM as the only site of metastasis may benefit from local therapies such as cytoreductive surgery and hyperthermic intraperitoneal chemotherapy (HIPEC; ref. 5). Nevertheless, the outcomes for patients with PM are variable. To refine treatment strategies, there is critical need for prognostic and predictive biomarkers that can help identify individuals who are most likely to derive benefits from aggressive local treatment interventions. Currently, the peritoneal cancer index, based on intraoperative assessment, is the only criterion suggested for the selection of patients with PM who may benefit from aggressive surgical treatment (6).

We hypothesized that patients with PM may have distinct characteristics that lead to different clinical outcomes compared with those without PM. Therefore, molecular characterization could offer insights into the clinical outcomes for patients with PM and allow for identification of unique biological drivers of peritoneal dissemination that can be targeted. Retrospective studies using single gene analysis or small targeted panel next-generation sequencing (NGS) have been performed in mCRC with PM (7–9). There is evidence that *BRAF* V600 mutations (10, 11) and microsatellite instability (MSI; ref. 12) are enriched in patients with PM. However, these studies often feature small sample sizes, lack a comparator group (non-PM mCRC) and include only limited genomic characterization. Project Genomics Evidence Neoplasia Information Exchange (GENIE) is an international genomics registry and data-sharing consortium launched in 2015 by the American Association of Cancer Research (AACR; ref. 13). Within GENIE, the BioPharma Collaborative (GENIE BPC) project augments the genomic data with comprehensive clinical data for cohorts of patients with selected cancers, including colorectal cancer. Our primary objective in this study was to characterize the genomic differences between patients with mCRC with and without PM. As secondary objectives, we examined the association between clinical characteristics and the presence of PM. In addition, we analysed the impact of PM and selected genomic alterations on overall survival (OS) from advanced disease and progression-free survival (PFS) from the initiation of first-line systemic therapy in the advanced setting.

## Materials and Methods

### Study Sample

Patients from the GENIE BPC colorectal cancer (CRC) v2.0-public data release were analyzed. These patients were selected for Project GENIE BPC based on the following eligibility criteria: underwent somatic genomic sequencing at one of three institutions (Dana-Farber Cancer Institute, Memorial Sloan Kettering Cancer Center, Vanderbilt-Ingram Cancer Center) between January 1, 2015 and April 30, 2018; aged 18 or older at the time of genomic sequencing; a minimum of 2 years of potential follow-up after sequencing; and one of the following OncoTree diagnoses: colorectal adenocarcinoma (COADREAD), colon adenocarcinoma (COAD), mucinous adenocarcinoma of the colon and rectum (MACR), signet ring cell adenocarcinoma of the colon and rectum (SRCCR), or rectal adenocarcinoma (READ). The PRISSMM (Pathology; Radiology; Imaging; Signs and Symptoms; tumor Markers; Medical oncology assessments) framework was used to curate structured clinical, demographic, treatment, pathology and imaging reports, and medical oncologist notes (14). The GENIE BPC project was approved by the research ethics board at each participating institution. Written informed consent was obtained for each patient to be included in this registry. Study was conducted in accordance to Declaration of Helsinki ethical guidelines.

Patients diagnosed with stage IV disease or stage I–III disease who later developed distant metastasis were evaluated. PM were defined as the presence of metastasis documented under any of the following ICD-O-3 (topography) codes on a cancer diagnosis form, radiology report, or pathology report at or after initial colorectal cancer diagnosis: C48.1 (specified parts of peritoneum), C48.2 (peritoneum not otherwise specified), C56.9 (ovary), C57.4 (uterine adnexa), and F20 (peritoneal fluid/ascites). Patients with PM at advanced diagnosis were further categorized into: (i) PM only, defined as those with PM as the only distant site disease present at the time of advanced diagnosis; and (ii) PM and other sites, defined as those with PM in addition to other locations of distant metastasis noted at the time of advanced disease diagnosis.

### Genomic Analysis

Each institution performed NGS analysis on archival formalin-fixed paraffin-embedded tissue from the primary tumor and/or metastases to detect single-nucleotide variants, small indels, copy-number alterations, and/or structural variants. Details regarding sequencing panels are provided in Supplementary Table S1 and in the GENIE data guide (www.aacr.org/wp-content/uploads/2022/02/GENIE_data_guide_11.0-public-1.pdf). In cases where a patient had multiple NGS reports available, only the first report was considered for analysis. A gene was considered altered in the presence of any mutation, fusion, amplification, or deletion. Genomic data were analyzed both with and without annotation for the OncoKB (precision Oncology Knowledge Base), such that the annotated genomic alterations were restricted to oncogenic and likely oncogenic variants.

Mismatch repair (MMR) status was determined on the basis of the pathology reports that indicated the expression of MLH1, MSH2, MSH6, or PMS2. Deficient MMR (dMMR) was defined as a loss of nuclear expression of at least one of these proteins. Patients were considered as dMMR if this status was ever documented on any available pathology report, while those with proficient MMR (pMMR) were identified if pMMR was recorded on any pathology report available. MMR non-concordance was assigned when both dMMR and pMMR were noted across pathology reports. For those tested for microsatellite instability (MSI) using PCR for microsatellites, the designation MSI-high (MSI-H) was assigned if MSI-H was recorded on any pathology report, and MSI-low/microsatellite stable (MSI-L/MSS) if either MSI-L or MSS was recorded on any pathology report. Patients were defined as MSI non-concordant if both MSI-H and MSI-L/MSS were recorded across pathology reports. Further details on the derivation of MMR and MSI statuses are shown in Supplementary Fig. S1.

### Statistical Analysis

The study cohort was summarized using descriptive statistics including frequencies, medians, and ranges. Associations between baseline clinical and pathologic characteristics with presence of PM were calculated using logistic regression. Multivariable analysis (MVA) for presence of PM included the variables that were significant in univariable analyses at a threshold of *P* < 0.05 and was adjusted for stage at diagnosis (IV vs. I–III), sex, primary tumor location, primary tumor histology, histologic grade of primary tumor, presence of liver metastasis at diagnosis of advanced disease (yes or no), and presence of lung metastasis at diagnosis of advanced disease (yes or no). Associations between genomic variables with PM were evaluated using the *χ*^2^ test or Fisher exact test. To control the false discovery rate (FDR) among comparisons of genomic alterations, adjustments were made using the Benjamini–Hochberg method in comparisons between genomic alterations and PM. A *q*-value < 0.05 was considered statistically significant.

We evaluated the associations between clinical and genomic variables of interest and time-to-event endpoints. OS was defined from diagnosis of advanced disease (either at diagnosis for stage IV or from date of distant metastasis among stage I–III) until death or last follow-up. OS calculations were restricted to patients known to be alive at the time of NGS report.

PFS was defined from initiation of the first combination drug therapy regimen received following an advanced diagnosis. First-received combination therapies included in the PFS cohort consisted of those recommended by the National Comprehensive Cancer Network: 5-fluorouracil, oxaliplatin and leucovorin (FOLFOX), 5-fluorouracil, irinotecan and leucovorin (FOLFIRI), 5-fluorouracil, oxaliplatin, irinotecan and leucovorin (FOLFOXIRI), or capecitabine and oxaliplatin (XELOX) ± Bevacizumab/Cetuximab. The PRISSMM framework defines four real-world PFS endpoints: (i) PFS-I (time to disease progression according to an imaging report, or death); (ii) PFS-M (time to disease progression according to a medical oncologist assessment, or death); (iii) PFS-I-or-M (time to disease progression according to the earlier of an imaging report or a medical oncologist assessment, or death); (iv) PFS-I-and-M (time to disease progression according to the later documentation of disease worsening that was recorded in both an imaging report and medical oncologist assessment, or death). All PFS endpoints censor patients without progression at the initiation of a subsequent drug regimen, if applicable, or last follow-up. In alignment with a previous analysis demonstrating that PFS-I-and-M correlates most strongly with OS (14), analyses of PFS-I-and-M are presented as the primary results, while the remaining three PFS analyses are provided in Supplementary Materials. All PFS calculations were restricted to patients who were alive and progression-free at the time of NGS report.

OS and PFS were estimated using Kaplan–Meier methodology. HRs along with 95% confidence intervals (CI) were calculated using the Cox proportional hazards models. All survival analyses account for the left truncation bias inherent to the data by using risk set adjustment methods (i.e., entering patients into the risk set at the time of NGS report; ref. 15). Because of the delayed entry, risk tables below Kaplan–Meier curves may show the number of patients at risk increasing over time. All Cox models were adjusted for time from diagnosis of advanced disease to NGS report to account for possible dependent left truncation. All covariates included in Cox models were assessed for proportional hazards using tests of weighted residuals (16). MVA for both OS and PFS included the variables that were significant in univariable setting at a threshold of *P* ≤ 0.05. MVA for OS was adjusted for the presence of PM at time of advanced disease (yes or no), stage at diagnosis (IV vs. I–III), any *RAS/BRAF* mutation (yes or no), any *APC* mutation (yes or no), presence of liver metastasis at diagnosis of advanced disease (yes or no), presence of lung metastasis at diagnosis of advanced disease (yes or no), and months from diagnosis of advanced disease to NGS report. MVA for PFS-I-and-M was adjusted for any *APC* mutation, presence of liver metastasis at advanced disease, and months from initiation of first-line drug regimen to NGS report. All *P* values were from two-sided tests and results were deemed statistically significant at *P* < 0.05.

Analyses were performed using R version 4.2.1. All genomic data were extracted from cBioPortal for cancer genomics using the *cbioportalR* R package, while all clinical data were extracted from Synapse using the *genieBPC* R package. Note that both *cbioportalR* and *genieBPC* are available on CRAN (17). This study has been performed and reported according to STROBE and REMARK guidelines from the Equator network.

### Data Availability Statement

All of the clinical and genomic data are publicly available on https://www.synapse.org/#!Synapse:syn30991637. To access the publicly available GENIE BPC data:

- Register for a *Synapse* account (https://www.synapse.org/#)
- Review and accept the *Synapse* account terms of use
- Navigate to the CRC v2.0-public data release (https://www.synapse.org/#!Synapse:syn30991637)
- Click “Request Access,” review the terms of data use, and select “Accept”
- All the R source codes are publicly available in the following link: https://github.com/slb2240/patients_with_peritoneal_metastasis_from_colorectal_cancer

## Results

### Clinical and Pathologic Characteristics

**FIGURE 1:**
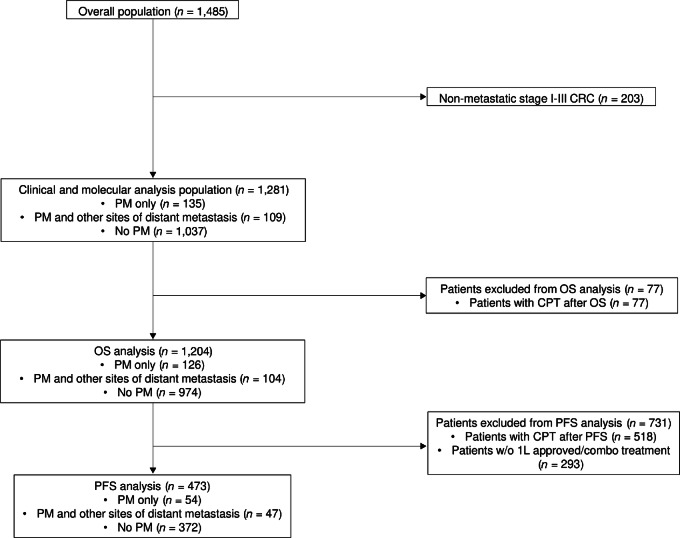
Flowchart of patient selection from GENIE BPC colorectal cancer registry.

A total of 1,485 patients were included in the GENIE BPC colorectal cancer cohort. The study cohort was comprised of the 1,281 patients with mCRC, including 700 patients diagnosed with stage IV colorectal cancer and 581 patients diagnosed with stage I–III colorectal cancer who later developed distant metastasis. PM were present in 244 patients, representing 16% of the entire GENIE colorectal cancer cohort and 19% of the study cohort (Fig. 1). Among these 244 patients with PM, 135 (55%) had PM only.

Clinical and demographic characteristics of the study population stratified by the presence of PM at advanced diagnosis are summarized in Table 1 and according to stage at diagnosis in Supplementary Table S2. The median age at diagnosis in the study cohort was 54 years [interquartile range (IQR): 47–64] and 55% were male. 77% (*N* = 982) of the patients in the study cohort were non-Hispanic White, while 7% (*N* = 91) of patients were non-Hispanic Black and 5% (*N* = 71) were Asian, Asian American, and Pacific Islander. Most cases were adenocarcinoma (*N* = 996, 91%) and 58% of patients had liver metastases present at time of advanced diagnosis (*N* = 723).

**TABLE 1:** Clinical and pathologic characteristics of the study cohort

Characteristic	Overall, *N* = 1,281^a^	No peritoneal metastasis, *N* = 1,037^a^	Peritoneal metastasis, *N* = 244^a^
Metastatic cohort			
Stage I–III with distant metastasis	581 (45%)	486 (47%)	95 (39%)
Stage IV	700 (55%)	551 (53%)	149 (61%)
Age at diagnosis (IQR)	54 (47–64)	55 (47–64)	52 (46–63)
Sex			
Female	571 (45%)	432 (42%)	139 (57%)
Male	710 (55%)	605 (58%)	105 (43%)
Race/ethnicity			
Non-Hispanic White	982 (77%)	801 (77%)	181 (74%)
Non-Hispanic Black	91 (7%)	75 (7%)	16 (7%)
AAAPI (Asian, Asian American, and Pacific Islander)	71 (5%)	55 (5%)	16 (6%)
Unknown race	59 (5%)	47 (5%)	12 (5%)
Hispanic/Latinx	50 (4%)	40 (4%)	10 (4%)
Other	28 (2%)	19 (2%)	9 (4%)
**Primary tumor**			
Location of primary tumor			
Left colon	388 (32%)	309 (31%)	79 (35%)
Rectal	431 (35%)	389 (39%)	42 (19%)
Right colon	398 (33%)	292 (29%)	106 (47%)
Unknown	64	47	17
Side of primary tumor			
Left	819 (67%)	698 (71%)	121 (53%)
Right	398 (33%)	292 (29%)	106 (47%)
Unknown	64	47	17
Histologic grade of primary tumor			
I/II	812 (74%)	690 (77%)	122 (61%)
III/IV	285 (26%)	206 (23%)	79 (39%)
Unknown	184	141	43
Primary tumor histology			
Adenocarcinoma	996 (91%)	831 (94%)	165 (79%)
Mucinous adenocarcinoma	67 (6.1%)	34 (3.8%)	33 (16%)
Other histologies/mixed tumor	18 (1.6%)	16 (1.8%)	2 (1.0%)
Signet ring cell carcinoma	16 (1.5%)	7 (0.8%)	9 (4.3%)
Unknown	184	149	35
MSI status			
MSI-H	21 (2%)	15 (1%)	5 (2%)
MSI-L/MSS	258 (20%)	203 (20%)	55 (23%)
MSI non-concordant	1 (<1%)	0 (0%)	1 (<1%)
Unknown MSI status	1,002 (78%)	819 (79%)	183 (75%)
MMR status			
dMMR	62 (5%)	47 (4%)	15 (6%)
pMMR	875 (68%)	704 (68%)	171 (70%)
MMR non-concordant	13 (1%)	10 (1%)	3 (1%)
Unknown MMR status	331 (26%)	276 (27%)	55 (23%)
**Sites of initial metastases (at the time of diagnosis with either stage IV disease or presentation with metastases following stage I–III)**			
Liver metastases	723 (58%)	660 (65%)	63 (26%)
Unknown	26	26	0
Lung metastases	266 (21%)	237 (23%)	29 (12%)
Unknown	26	26	0
Brain metastases	8 (0.6%)	8 (0.8%)	0 (0%)
Unknown	26	26	0
Bone metastases	35 (2.8%)	29 (2.9%)	6 (2.5%)
Unknown	26	26	0
NGS report returned after death or the date of last follow-up	77 (6.0%)	63 (6.1%)	14 (5.7%)

Abbreviations: dMMR, deficient mismatch repair system; IQR, interquartile range; MSI-H, MSI-high; MSI-L/MSS, MSI-low/microsatellite stable; pMMR, proficient mismatch repair system.

^a^ *n* (%); Median (IQR).

Associations between clinical and pathologic characteristics with PM are shown in Table 2. In a multivariable model for presence of PM at diagnosis of advanced disease, PM were associated with female sex (OR: 1.67; 95% CI: 1.11–2.54; *P* = 0.014) and high histologic grade (OR: 1.72; 95% CI: 1.08–2.71; *P* = 0.002) after adjustment for other independently significant clinical variables. Tumor location (left colon vs. right colon vs. rectum) was associated with presence of PM (rectal vs. left colon OR: 0.51, 95% CI: 0.29–0.88; right colon vs. left colon OR: 1.41, 95% CI: 0.86–2.32; *P* < 0.001). Presence of liver metastasis and lung metastasis were less frequent in patients with PM compared with non-PM (liver OR: 0.04, 95% CI: 0.03–0.08, *P* < 0.001; lung OR: 0.33, 95% CI: 0.18–0.58, *P* < 0.001). Histology was associated with presence of PM (*P* = 0.001), with mucinous adenocarcinoma occurring more frequently than adenocarcinoma in patients with PM (OR: 3.55; 95% CI: 1.72–7.42).

**TABLE 2:** Univariable and multivariable logistic regression models for associations between baseline characteristics and the presence of PM

*Univariable logistic regression models*			
Characteristic	OR	95% CI	*P*-value
Metastatic cohort			**0.025**
Stage I–III with distant metastasis	—	—	
Stage IV	1.38	1.04–1.84	
Age at diagnosis	0.99	0.98–1.00	0.077
Sex			**<0.001**
Male	—	—	
Female	1.85	1.40–2.46	
Race/ethnicity			0.6
Non-Hispanic White	—	—	
AAAPI (Asian, Asian American, and Pacific Islander)	1.29	0.70–2.25	
Hispanic/Latinx	1.11	0.51–2.17	
Non-Hispanic Black	0.94	0.52–1.62	
Other	2.10	0.89–4.59	
Unknown	1.13	0.56–2.11	
Location of primary tumor			**<0.001**
Left colon	—	—	
Rectal	0.42	0.28–0.63	
Right colon	1.42	1.02–1.98	
Side of primary tumor			**<0.001**
Left	—	—	
Right	2.09	1.56–2.81	
Histologic grade of primary tumor			**<0.001**
I/II	—	—	
III/IV	2.17	1.57–2.99	
Primary tumor histology			**<0.001**
Adenocarcinoma	—	—	
Mucinous adenocarcinoma	4.89	2.94–8.13	
Other	0.63	0.10–2.24	
Signet ring cell carcinoma	6.48	2.38–18.4	
MSI status			0.7
MSI-L/MSS	—	—	
MSI-H	1.23	0.39–3.33	
MMR status			0.7
pMMR	—	—	
dMMR	1.31	0.70–2.35	
MMR non-concordant	1.24	0.27–4.09	
Lung metastases at advanced diagnosis	0.44	0.29–0.66	**<0.001**
Liver metastases at advanced diagnosis	0.19	0.13–0.25	**<0.001**
Bone metastases at advanced diagnosis	0.85	0.32–1.94	0.7
***Multivariable logistic regression models***			
**Characteristic**	**OR**	**95% CI**	***P*-value**
Metastatic cohort			**<0.001**
Stage I–III with distant metastasis	—	—	
Stage IV	6.54	3.91–11.30	
Sex			**0.014**
Male	—	—	
Female	1.67	1.11–2.54	
Location of primary tumor			**<0.001**
Left colon	—	—	
Rectal	0.51	0.29–0.88	
Right colon	1.41	0.86–2.32	
Histologic grade of primary tumor			**0.022**
I/II	—	—	
III/IV	1.72	1.08–2.71	
Primary tumor histology			**0.001**
Adenocarcinoma	—	—	
Mucinous adenocarcinoma	3.55	1.72–7.42	
Other	0.74	0.04–5.15	
Signet ring cell carcinoma	4.58	1.23–17.20	
Lung metastases at advanced diagnosis	0.33	0.18–0.58	**<0.001**
Liver metastases at advanced diagnosis	0.04	0.03–0.08	**<0.001**

NOTE: Variables with univariable *P* values < 0.05 were included in the multivariable model. One patient with MSI non-concordant status was omitted from univariable analysis.

Abbreviations: CI, confidence interval; dMMR, deficient mismatch repair system; MSI-H, MSI-high; MSI-L/MSS, MSI-low/microsatellite stable; OR, odds ratio; pMMR, proficient mismatch repair system.

### Association Between Molecular Characteristics and PM

A total of 1,345 NGS reports were available for the 1,281 patients included in the study cohort. 62% (*N* = 792) of patients’ NGS samples were taken from the primary tumor, with a median time interval from advanced diagnosis to NGS report of 7 months (IQR: 2–20). The site of NGS sample (primary tumor vs. metastasis) and type of NGS panel are summarized in Supplementary Table S3. Genes of particular clinical interest, *NRAS* and *MED12,* as well as frequencies of all genes altered in at least 7% of the samples and tested in at least 20% of the samples*,* are presented in Table 3 and are visualized by presence of PM in Supplementary Fig. S2 and S3. The five most frequently altered genes were *APC* (*N* = 151, 64% vs. *N* = 788, 79%), *TP53* (*N* = 167, 68% vs. *N* = 779, 75%), *KRAS* (*N* = 122, 50% vs. *N* = 457, 44%), *PIK3CA* (*N* = 50, 20% vs. *N* = 458, 21%), and *SMAD4* (*N* = 50, 21% vs. *N* = 176, 18%) in PM and non-PM patients, respectively. These frequencies were similar when restricting to only oncogenic and likely oncogenic variants according to OncoKB annotation.

**TABLE 3:** Frequencies of genomic alterations in tumor samples—Results are displayed for genomic alterations present in at least 7% of tumor specimens and tested in at least 20% of the tumor specimens, as well as *NRAS* and *MED12.* Frequencies do not reflect OncoKB annotation. Details of specific NGS panels performed can be found in the GENIE data guide (www.aacr.org/wp-content/uploads/2022/02/GENIE_data_guide_11.0-public-1.pdf)

Characteristic	Overall, *N* = 1,281^a^	No peritoneal metastasis, *N* = 1,037^a^	Peritoneal metastasis, *N* = 244^a^	*P*-value	*q*-value^b^
*APC*	939 (76%)	788 (79%)	151 (64%)	<0.001^c^	<0.001
Unknown	52	44	8		
*TP53*	946 (74%)	779 (75%)	167 (68%)	0.033^c^	0.3
*KRAS*	579 (45%)	457 (44%)	122 (50%)	0.094^c^	0.4
*PIK3CA*	266 (21%)	216 (21%)	50 (20%)	>0.9^c^	>0.9
*SMAD4*	226 (18%)	176 (18%)	50 (21%)	0.2^c^	0.5
Unknown	52	44	8		
*FBXW7*	154 (13%)	124 (12%)	30 (13%)	>0.9^c^	>0.9
Unknown	52	44	8		
*SOX9*	144 (12%)	117 (12%)	27 (11%)	0.9^c^	>0.9
Unknown	53	45	8		
*KMT2D*	143 (12%)	112 (11%)	31 (13%)	0.4^c^	0.7
Unknown	52	44	8		
*BRAF*	138 (11%)	108 (10%)	30 (12%)	0.4^c^	0.7
*TCF7L2*	111 (11%)	97 (11%)	14 (7.0%)	0.075^c^	0.4
Unknown	226	181	45		
*ARID1A*	124 (10%)	99 (10.0%)	25 (11%)	0.8^c^	>0.9
Unknown	52	44	8		
*PTPRT*	59 (9.9%)	49 (10%)	10 (9.2%)	0.8^c^	>0.9
Unknown	684	549	135		
*PRKDC*	59 (9.3%)	46 (9.1%)	13 (10%)	0.7^c^	>0.9
Unknown	649	532	117		
*ATM*	110 (9.0%)	88 (8.9%)	22 (9.3%)	0.8^c^	>0.9
Unknown	52	44	8		
*PTPRS*	53 (8.9%)	42 (8.6%)	11 (10%)	0.6^c^	0.9
Unknown	684	549	135		
*ASXL1*	104 (8.5%)	88 (8.9%)	16 (6.8%)	0.3^c^	0.6
Unknown	52	44	8		
*GLI2*	39 (8.4%)	35 (9.3%)	4 (4.4%)	0.13^c^	0.4
Unknown	816	662	154		
*BRCA2*	103 (8.4%)	87 (8.8%)	16 (6.8%)	0.3^c^	0.6
Unknown	52	44	8		
*FAT1*	79 (8.0%)	60 (7.6%)	19 (9.6%)	0.3^c^	0.6
Unknown	294	247	47		
*RTEL1*	23 (7.7%)	16 (6.5%)	7 (13%)	0.2^d^	0.4
Unknown	981	792	189		
*AMER1*	58 (7.6%)	46 (7.4%)	12 (8.2%)	0.8^c^	>0.9
Unknown	517	419	98		
*RNF43*	75 (7.6%)	59 (7.5%)	16 (8.1%)	0.8^c^	>0.9
Unknown	293	246	47		
*PREX2*	35 (7.5%)	31 (8.3%)	4 (4.3%)	0.2^c^	0.5
Unknown	815	663	152		
*ARID1B*	92 (7.5%)	69 (7.0%)	23 (9.7%)	0.14^c^	0.4
Unknown	53	45	8		
*NOTCH3*	73 (7.4%)	60 (7.6%)	13 (6.6%)	0.6^c^	0.9
Unknown	293	246	47		
*NOTCH1*	94 (7.3%)	66 (6.4%)	28 (11%)	0.006^c^	0.061
*CREBBP*	90 (7.3%)	68 (6.8%)	22 (9.3%)	0.2^c^	0.5
Unknown	52	44	8		
*FLT1*	89 (7.2%)	74 (7.5%)	15 (6.4%)	0.6^c^	0.9
Unknown	52	44	8		
*FLT3*	88 (7.2%)	77 (7.8%)	11 (4.7%)	0.10^c^	0.4
Unknown	52	44	8		
*MED12*	52 (5.3%)	32 (4.0%)	20 (10%)	<0.001^c^	0.009
Unknown	293	246	47		
*NRAS*	69 (5.4%)	61 (5.9%)	8 (3.3%)	0.11^c^	0.4

NOTE: Estimations of genomic alterations presented are not annotated according to OncoKB.

^a^ *n* (%).

^b^FDR correction for multiple testing.

^c^Pearson *χ*^2^ test.

^d^Fisher exact test.

Three genes with direct impact in upfront therapy selection for mCRC (*KRAS*, *NRAS,* and *BRAF)* were sequenced on all NGS panels. There were no differences in frequency of mutations in these three genes between PM and without PM (Table 3), nor was there any difference in the presence of *BRAF* V600E mutation between PM and non-PM (9.8% vs. 6.6%, *P* = 0.10). After controlling for the FDR, the only significant differences in genomic alterations between PM only versus PM and other sites versus no PM were alterations in *APC* and *MED12.* APC was less frequently altered in patients with PM (60%) compared with patients with PM and other sites (68%) and non-PM (79%; *q* < 0.01). *MED12* was more frequently altered among patients with PM only (13%) compared with patients with PM and other sites of distant metastasis (7.4%) and patients without PM (4%; *q* = 0.02). These differences were also observed when comparing *MED12* and *APC* alterations between patients with PM versus no PM (Table 3). However, when restricting the analysis to oncogenic and likely oncogenic variants based on OncoKB annotation, there were no significant differences observed in the presence of *MED12* alterations when comparing patients with PM only versus PM and other sites versus no PM or between patients with PM versus without PM*. NOTCH1* alterations were observed more frequently in patients with PM versus without PM (11% vs. 6.4%) but these differences were not statistically significant after adjusting by multiple comparisons (*q*-value 0.06).

At least one of MSI status or MMR status was known in 82% of the patients (*N* = 1,049). MSI results were available for 22% of the study cohort (*N* = 279). Among patients with MSI results available, 7.5% were MSI-H (*N* = 21) and 4.3% were MSI-L/MSS (*N* = 258). MMR results were available in 958 patients (75%), and 5% (*N* = 62) of these were dMMR. There were no differences in MMR status between patients with and without PM (dMMR 6% vs. 4%, *P* = 0.7) in the entire study cohort, although among patients diagnosed at stage IV disease, dMMR was more frequent in patients with PM compared with non-PM (9% vs. 2%, *P* < 0.01; Supplementary Table S2).

### Survival Endpoints: OS

**FIGURE 2:**
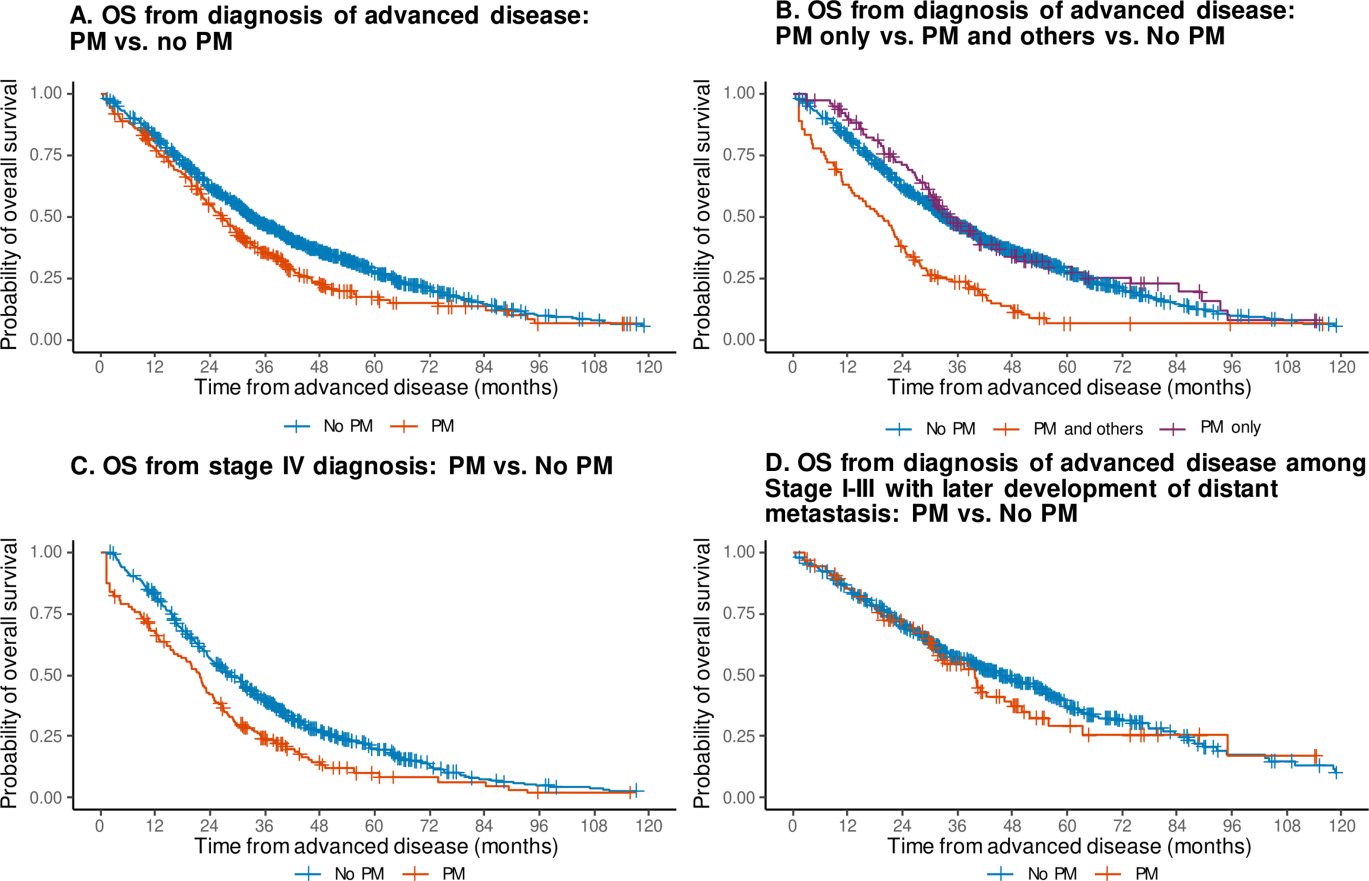
OS from diagnosis of advanced disease (**A,** PM vs. non-PM; **B,** PM only vs. PM and other sites vs. non-PM; **C,** stage IV: PM vs. non-PM; **D,** stage I–III with later development of distant metastasis: PM vs. non-PM).

The cohort evaluable for OS included 1,204 patients after excluding 77 patients from the study cohort whose NGS report was returned after the last follow-up visit or death (Fig. 1). Baseline characteristics of this population are summarized in Supplementary Table S4. OS estimates according to PM and stage at diagnosis are summarized in Fig. 2. The median OS from the diagnosis of advanced disease was 31.4 months (95% CI: 28.9–34.2). OS was worse in patients with PM compared with those without PM [median OS: 26.8 months (95% CI: 22.2–31.3) vs. 32.9 months (95% CI: 30.4–37.7), *P* < 0.01] (Fig. 2A). After stratifying by stage at diagnosis, this difference was still observed among patients diagnosed with stage IV disease (*P* = 0.02), but not among patients diagnosed with stage I–III disease who later developed metastasis (*P* = 0.42; Fig. 2C and D).

OS from advanced disease was significantly shorter for patients with PM and other sites of metastases (median OS: 19.6 months, 95% CI: 10.6–24.8) compared with those with PM only (median OS: 34.3 months, 95% CI: 29.9–44.0) and those without PM but with other sites of metastases (median OS: 32.9 months, 95% CI: 30.4–37.7; *P* < 0.01; Fig. 2B). Stratification by stage at diagnosis demonstrated that this difference was maintained for patients diagnosed with stage IV disease (*P* < 0.01), but not for patients diagnosed with stage I–III colorectal cancer who later developed metastasis (*P* = 0.17).

We sought to explore whether there was an impact on OS based on the presence of PM and the status of *KRAS, BRAF, NRAS,* any of *RAS/BRAF,* or three known driver mutations in colorectal cancer: *TP53, APC,* and *PIK3CA* (Supplementary Table S5). OS was significantly different for all four analyses of presence of PM by *KRAS* (*P* < 0.01), *BRAF* (*P* = 0.04), *NRAS* (*P* = 0.04), and *RAS/BRAF* (*P* < 0.01) mutant (mt) versus wild-type (wt) patients, with the longest median OS in the non-PM wt subgroups compared with the non-PM mt, PM wt, and PM mt subgroups. In contrast, in the analyses of presence of PM by *TP53, APC,* and *PIK3CA* alteration statuses, OS differed significantly among the subgroups with non-PM mt patients having the longest estimated median OS (*TP53: P* = 0.03; *APC: P* < 0.01; *PIK3CA: P* = 0.01) compared with the non-PM wt, PM mt, and PM wt subgroups. These associations remained statistically significant when limited to oncogenic and likely oncogenic variants according to OncoKB.

### Univariable and Multivariable Analysis for OS

Univariable analyses were conducted to examine associations between clinical and genomic factors of interest with OS (Table 4). Among the clinical factors, univariable analyses showed that stage at diagnosis (stage IV vs. stage I–III) and the presence of PM were associated with worse OS [stage HR: 1.80 (95% CI: 1.54–2.12), *P* < 0.001; PM HR: 1.30 (95% CI: 1.07–1.57), *P* < 0.01]. In addition, the presence of liver metastasis and lung metastasis at advanced diagnosis were associated with worse OS [liver metastasis HR: 1.43 (95% CI: 1.21–1.68), *P* < 0.01; lung metastasis HR: 1.24 (95% CI: 1.03–1.49), *P* = 0.02]. No significant OS differences were observed between location of primary tumor (right colon vs. left colon vs. rectum; *P* = 0.3), MSI status (MSI-H vs. MSI-L/MSS; *P* = 0.4), or MMR status (pMMR vs. dMMR vs. MMR non-concordant; *P* = 0.065). Beyond the clinical variables, we explored associations between OS and *RAF/BRAF, TP53, APC,* and *PIK3CA* alteration status*.* In the univariable setting, the presence of any *RAS/BRAF* alteration was associated with worse OS [HR: 1.53 (95% CI: 1.31–1.80), *P* < 0.01]. There were no differences in OS by the presence of *TP53* or *PIK3CA* alterations, but the presence of *APC* alterations was associated with longer OS under univariable analysis [HR: 0.75 (95% CI: 0.63–0.90), *P* < 0.01]. Similar associations with OS were observed when restricting to oncogenic and likely oncogenic variants.

**TABLE 4:** Univariable and multivariable Cox proportional hazards models for OS

*Univariable Cox proportional hazards models*					
Characteristic	*N*	Event *N*	HR	95% CI	*P*-value
Presence of peritoneal metastasis					**0.008**
No peritoneal metastasis	973	519	—	—	
Peritoneal metastasis	230	137	1.30	1.07–1.57	
Presence of peritoneal metastasis					**<0.001**
No peritoneal metastasis	973	519	—	—	
Peritoneal metastasis and other sites of distant metastasis	104	74	1.86	1.45–2.37	
Peritoneal metastasis only	126	63	0.96	0.74–1.25	
Metastatic cohort					**<0.001**
Stage I–III with distant metastases	548	233	—	—	
Stage IV	655	423	1.80	1.54–2.12	
Location of primary tumor					0.3
Left colon	368	192	—	—	
Rectum	406	217	1.07	0.88–1.29	
Right colon	369	205	1.18	0.97–1.44	
MSI status					0.4
MSI-L/MSS	240	144	—	—	
MSI-H	16	8	0.74	0.36–1.52	
MMR status					0.065
pMMR	824	449	—	—	
dMMR	57	23	0.69	0.45–1.05	
MMR non-concordant	12	4	0.51	0.19–1.36	
Any *KRAS/BRAF/NRAS* alteration	693	410	1.53	1.31–1.80	**<0.001**
*APC* alteration	881	459	0.75	0.63–0.90	**0.002**
*TP53* alteration	875	472	0.97	0.82–1.15	0.7
*PIK3CA* alteration	251	123	0.85	0.70–1.03	0.090
Liver metastases at advanced diagnosis	684	414	1.43	1.21–1.68	**<0.001**
Lung metastases at advanced diagnosis	249	149	1.24	1.03–1.49	**0.024**
***Multivariable Cox proportional hazards model***					
**Characteristic**			**HR**	**95% CI**	***P*-value**
Time (months) from advanced diagnosis to NGS report	1,129	611	1.01	1.01–1.02	**<0.001**
Presence of peritoneal metastasis					**0.001**
No peritoneal metastasis	906	479	—	—	
Peritoneal metastasis	223	132	1.45	1.16–1.81	
Metastatic cohort					**<0.001**
Stage I–III with distant metastases	521	218	—	—	
Stage IV	608	393	1.56	1.28–1.89	
Any *KRAS/BRAF/NRAS* alteration	658	386	1.52	1.28–1.79	**<0.001**
*APC* alteration	866	452	0.77	0.64–0.93	**0.009**
Liver metastases at advanced diagnosis	650	394	1.46	1.18–1.80	**<0.001**
Lung metastases at advanced diagnosis	240	144	1.37	1.13–1.67	**0.002**

Abbreviations: CI, confidence interval; dMMR, deficient mismatch repair system; HR, hazard ratio; MSI-H, MSI-high; MSI-L/MSS, MSI-low/microsatellite stable; pMMR, proficient mismatch repair system.

NOTE: Variables with univariable *P* values < 0.05 were included in the multivariable model. One patient with MSI non-concordant status was omitted from univariable analysis. The “Any *KRAS/BRAF/NRAS* alteration” variable did not meet the proportional hazards assumption for the multivariable model but was retained because of clinical importance. Estimations of genomic alterations presented are not annotated according to OncoKB.

MVA demonstrated that the presence of PM at advanced diagnosis was an independent prognostic factor for worse OS [HR: 1.45 (95% CI: 1.16–1.81), *P* < 0.01] after adjustment for stage at diagnosis, *RAS/BRAF* and *APC* alteration status, presence of liver metastasis and presence of lung metastasis at diagnosis of advanced disease, and months from diagnosis of advanced disease to NGS report (Table 4).

### Survival Endpoints: PFS

**FIGURE 3:**
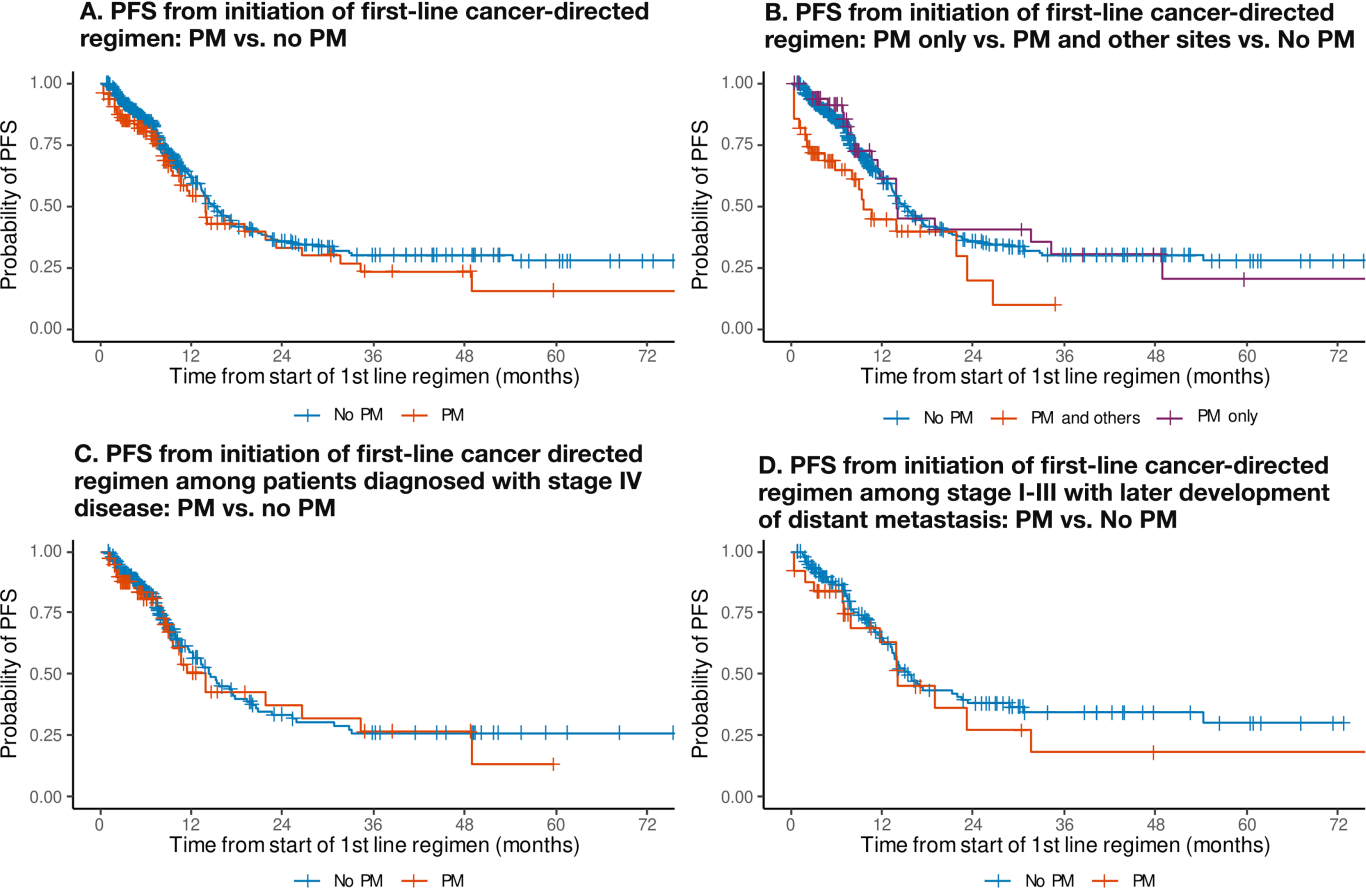
PFS from initiation of first-line cancer-directed regimen (**A,** PM vs. non-PM; **B,** PM only vs. PM and other sites vs. non-PM; **C,** stage IV: PM only vs. PM and other sites vs. non-PM; **D,** stage I–III with later development of distant metastasis: PM vs. non-PM).

There were 473 patients who received first-line approved combination drug therapies and were included in the PFS-I-and-M cohort (101 patients with PM, 21.4%; Fig. 1). Most patients received a combination of fluoropyrimidines and oxaliplatin as their first-line systemic therapy (69%, Supplementary Table S6). PFS-I-and-M according to PM and stage are summarized in Fig. 3. There was no significant difference in PFS-I-and-M from initiation of first-line therapy when comparing patients with PM versus without PM [median PFS: 13.8 months (95% CI: 10.6–26.5) vs. 14.9 months (95% CI: 13.3–17.7), respectively; *P* value = 0.46] (Fig. 3A). Stratification by stage at diagnosis revealed no significant differences in comparisons of PM versus non-PM and PFS-I-and-M (Fig. 3C and D). Similarly, there was no difference in PFS-I-and-M when comparing among patients with PM only versus PM and other sites versus non-PM [median PFS-I-and-M: 13.9 months (95% CI: 11.7–not reached) vs. 9.5 months (95% CI: 4.4–not reached) vs. 14.9 months (95% CI: 13.3–17.7); *P* = 0.40] (Fig. 3B). Secondary analyses estimating the other PRISSMM-defined PFS endpoints (PFS-I, PFS-M, and PFS-I-or-M) by presence of PM are presented in Supplementary Table S7.

We examined whether there was an impact on PFS-I-and-M based in the presence of PM and *KRAS, BRAF, RAS/BRAF, PIK3CA, APC,* and *TP53* mutation status (Supplementary Table S8). None of the comparisons revealed any significant differences in PFS-I-and-M based in the presence of PM and alterations. Note that the impact of *NRAS* by the presence of PM on PFS-I-and-M could not be explored because of limited subgroup sample sizes.

### Univariable and Multivariable Analysis for PFS

Univariable analyses of the associations between clinical and genomic variables and PFS-I-and-M were examined (Supplementary Table S9). Presence of liver metastasis at advanced diagnosis was the only clinical variable marginally associated with worse PFS-I-and-M [HR: 1.37, (95% CI: 1.00–1.88), *P* = 0.05]. Notably, neither the presence of PM (PM vs. non-PM) nor PM only versus PM and other sites of distant metastasis versus non-PM were significantly associated with PFS-I-and-M (*P* = 0.5 and *P* = 0.4, respectively). With respect to the genomic variables, the presence of *APC* alteration was significantly associated with better PFS-I-and-M [HR: 0.69 (95% CI: 0.48–0.98), *P* = 0.04]. However, this association dissipated after limiting to only oncogenic and likely oncogenic variants as per OncoKB annotation (*P* = 0.07).

MVA for PFS-I-and-M demonstrated that *APC* was significantly associated with better PFS-I-and-M after adjusting for the presence of liver metastasis at advanced diagnosis and time from advanced diagnosis to NGS report [HR: 0.61 (95% CI: 0.42–0.89), *P* = 0.01]. In addition, liver metastasis at diagnosis of advanced disease was significantly associated with worse PFS-I-and-M after adjusting for *APC* mutation status and time to NGS report.

## Discussion

The aim of this study was to characterize and compare the clinical and genomic characteristics of patients with mCRC with versus without PM. Consistent with prior large studies, PM were present in 19% of patients at diagnosis of advanced disease (18). The presence of PM was associated with several clinical factors that have been identified in prior series, including female sex (2), high histologic grade (19, 20), and mucinous or signet cell histology (21, 22). Most tumors (95%) were sequenced with large (326–468 genes) targeted tumor sequencing panels.

We identified two important genomic findings in patients with mCRC with PM. First, the presence of *MED12* alterations was associated with PM. Previously described as a tumor suppressor in colorectal cancer, *MED12* encodes a component of the mediator transcription regulation complex that is necessary for the initiation of the transcription (23, 24). When *MED12* is mutated or its expression is lost, it may induce an epithelial/mesenchymal-like phenotype and activation of the TGF receptor pathway that confer drug resistance in colorectal cancer models (25). This provides a therapeutic rationale to test TGFβ pathway targeted drugs in patients with *MED12* alterations or loss of expression. However, this association was not observed when variants were annotated by OncoKB, as most *MED12* alterations were non-oncogenic or of unknown significance because variants in this gene have not been well biologically characterized. In contrast, *APC* alterations were less frequent in patients with PM in the study cohort, even after restricting for oncogenic and likely oncogenic variants, supporting a key finding from a prior study that demonstrated that *APC* mutations were more frequent in primary colorectal cancer tumors compared with unmatched PM samples (26). *APC* is a key negative regulator of the Wnt pathway, which controls the cell proliferation and dedifferentiation of the gastrointestinal tract (27). In addition, *APC* alterations can contribute to the loss of cell adhesion and errors in cell cycle control or in DNA repair (28). Gene expression analysis has also shown that PM are associated with activation of the WNT/β-catenin pathway that can be activated because of other genomic alterations beyond *APC* (29). Therefore, WNT/β-catenin pathway may be more frequently activated in mCRC with PM. Moreover, certain *MED12* variants induce WNT/β-catenin pathway activation in myometrial cells (30, 31). This provides a rationale for targeting the WNT/β-catenin pathway in patients with PM.

In comparison with a recent systematic review, we observed a higher prevalence of mutations in *TP53* (68% vs. 54%) and *APC* (63% vs. 44%) and a similar prevalence of *KRAS* alterations (50% vs. 44%) in patients with mCRC with PM (8). In contrast to prior reports, *BRAF* was not associated with PM (10, 11, 32). Notably, this study cohort captures *BRAF* mutations beyond the canonical V600 hotspot analyzed in previous studies. When considering *BRAF* V600E canonical mutations only, such alterations did not show an increased frequency in patients with PM compared with no PM (9.8% vs. 6.6%).

Patients with PM had shorter OS from advanced disease compared with patients without PM as described previously (2). Notably, patients with PM and other sites of distant metastasis present at the time of advanced diagnosis had worse OS than patients with PM only and patients without PM. After adjustment for stage at diagnosis, *RAS/BRAF* and *APC* alteration statuses, presence of liver and lung metastases, and time from diagnosis of advanced disease to NGS report, patients with any PM had significantly shorter OS than those without PM, indicating that PM are an independent negative prognostic factor for patients with mCRC. *KRAS* and *BRAF* mutations are associated with shorter survival in mCRC irrespective of the presence of PM (33, 34). We observed in our cohort that patients with PM and *KRAS* mutation showed the worst prognosis. There were no differences in PFS-I-and-M from initiation of combination first-line cancer-directed drug regimens between patients with versus without PM or among patients with PM only versus PM and other sites of metastasis versus no PM. Sensitivity analyses of PFS-I, PFS-M, and PFS-I-or-M demonstrated similar patterns to the PFS-I-and-M results, with patients with PM generally having shorter median PFS estimates than those without PM, and patients with PM and other sites of metastasis with shorter median PFS compared with those with PM only and non-PM, though the differences were not statistically significant.

There are several limitations of this retrospective study. Comprised of data from three large academic medical institutions in the United States, this study may not accurately reflect the outcomes of patients with PM treated outside such centers specializing in management of mCRC. MSI status was largely missing within the study cohort but many other samples were tested instead for MMR protein status, that is usually performed as an alternative to MSI analysis. In addition, PM were identified using different ICD-O-3 codes that were abstracted by the curators in the GENIE BPC project. Therefore, some patients with PM at diagnosis may not have been identified if the sites of metastatic disease were not fully captured in the clinical notes, imaging or pathology reports during the initial diagnosis. Furthermore, clinical data on peritoneal surgery were not available, which could potentially influence the survival results. Although all patients had NGS testing, there were differences in the genes included in these assays. Our analysis of genomic alterations associated with PM accounts for these differences in gene coverage by restricting analyses of genomic alterations to tumors with the gene(s) of interest included on the sequencing panel. Finally, analysis was performed with the first available NGS testing when more than one sample was available, and it is possible that genomic alterations were acquired over the course of treatment.

In conclusion, this study demonstrates that patients with mCRC with PM have distinct clinical and molecular characteristics compared with those without PM, including differences in histologic grade, tumor location, and presence of *MED12* and *APC* alterations. To our knowledge, this is the largest multicenter, clinico-genomic study to evaluate the impact of PM on patients with colorectal cancer. Further research is needed to understand these biological differences and develop therapeutic strategies to prevent and treat mCRC with PM.

## Supplementary Material

Supplementary Figure 1 Definitions of patient-level MSI and MMR statuses

Supplementary Figure 2 Supplementary Figure 2. Oncoprint from patients without peritoneal metastasis

Supplementary Figure 3 Oncoprint of patients with peritoneal metastasis

Supplementary Tables Supplementary Tables 1-9

## References

[1] Siegel RL , Miller KD, Fuchs HE, Jemal A. Cancer statistics, 2021. CA Cancer J Clin 2021;71:7–33. 33433946 10.3322/caac.21654

[2] Franko J , Shi Q, Meyers JP, Maughan TS, Adams RA, Seymour MT, . Prognosis of patients with peritoneal metastatic colorectal cancer given systemic therapy: an analysis of individual patient data from prospective randomised trials from the Analysis and Research in Cancers of the Digestive System (ARCAD) database. Lancet Oncol 2016;17:1709–19. 27743922 10.1016/S1470-2045(16)30500-9

[3] Kranenburg O , van der Speeten K, de Hingh I. Peritoneal metastases from colorectal cancer: defining and addressing the challenges. Front Oncol 2021;11:650098. 33816304 10.3389/fonc.2021.650098 PMC8010649

[4] Van Cutsem E , Cervantes A, Adam R, Sobrero A, Van Krieken JH, Aderka D, . ESMO consensus guidelines for the management of patients with metastatic colorectal cancer. Ann Oncol 2016;27:1386–422. 27380959 10.1093/annonc/mdw235

[5] Turaga K , Levine E, Barone R, Sticca R, Petrelli N, Lambert L, . Consensus guidelines from The American Society of Peritoneal Surface Malignancies on standardizing the delivery of hyperthermic intraperitoneal chemotherapy (HIPEC) in colorectal cancer patients in the United States. Ann Surg Oncol 2014;21:1501–5. 23793364 10.1245/s10434-013-3061-z

[6] Elias D , Mariani A, Cloutier AS, Blot F, Goere D, Dumont F, . Modified selection criteria for complete cytoreductive surgery plus HIPEC based on peritoneal cancer index and small bowel involvement for peritoneal carcinomatosis of colorectal origin. Eur J Surg Oncol 2014;40:1467–73. 25086990 10.1016/j.ejso.2014.06.006

[7] Graf W , Cashin PH, Ghanipour L, Enblad M, Botling J, Terman A, . Prognostic impact of BRAF and KRAS mutation in patients with colorectal and appendiceal peritoneal metastases scheduled for CRS and HIPEC. Ann Surg Oncol 2020;27:293–300. 31571052 10.1245/s10434-019-07452-2 PMC6925063

[8] Lund-Andersen C , Torgunrud A, Fleten KG, Flatmark K. Omics analyses in peritoneal metastasis-utility in the management of peritoneal metastases from colorectal cancer and pseudomyxoma peritonei: a narrative review. J Gastrointest Oncol 2021;12:S191–203. 33968437 10.21037/jgo-20-136 PMC8100703

[9] Yaeger R , Chatila WK, Lipsyc MD, Hechtman JF, Cercek A, Sanchez-Vega F, . Clinical sequencing defines the genomic landscape of metastatic colorectal cancer. Cancer Cell 2018;33:125–36. 29316426 10.1016/j.ccell.2017.12.004 PMC5765991

[10] Sasaki Y , Hamaguchi T, Yamada Y, Takahashi N, Shoji H, Honma Y, . Value of KRAS, BRAF, and PIK3CA mutations and survival benefit from systemic chemotherapy in colorectal peritoneal carcinomatosis. Asian Pac J Cancer Prev 2016;17:539–43. 26925640 10.7314/apjcp.2016.17.2.539

[11] Prasanna T , Karapetis CS, Roder D, Tie J, Padbury R, Price T, . The survival outcome of patients with metastatic colorectal cancer based on the site of metastases and the impact of molecular markers and site of primary cancer on metastatic pattern. Acta Oncol 2018;57:1438–44. 30035653 10.1080/0284186X.2018.1487581

[12] Kim CG , Ahn JB, Jung M, Beom SH, Kim C, Kim JH, . Effects of microsatellite instability on recurrence patterns and outcomes in colorectal cancers. Br J Cancer 2016;115:25–33. 27228287 10.1038/bjc.2016.161 PMC4931375

[13] AACR Project GENIE Consortium. AACR project GENIE: powering precision medicine through an international consortium. Cancer Discov 2017;7:818–31. 28572459 10.1158/2159-8290.CD-17-0151 PMC5611790

[14] Kehl KL , Riely GJ, Lepisto EM, Lavery JA, Warner JL, LeNoue-Newton ML, . Correlation between surrogate end points and overall survival in a multi-institutional clinicogenomic cohort of patients with non–small cell lung or colorectal cancer. JAMA Netw Open 2021;4:e2117547. 34309669 10.1001/jamanetworkopen.2021.17547 PMC8314138

[15] Brown S , Lavery JA, Shen R, Martin AS, Kehl KL, Sweeney SM, . Implications of selection bias due to delayed study entry in clinical genomic studies. JAMA Oncol 2022;8:287–91. 34734967 10.1001/jamaoncol.2021.5153 PMC9190030

[16] Grambsch PM , Therneau TM. Proportional hazards tests and diagnostics based on weighted residuals. Biometrika 1994;81:515–26.

[17] Lavery JA , Brown S, Curry MA, Martin A, Sjoberg DD, Whiting K. A data processing pipeline for the AACR project GENIE biopharma collaborative data with the {genieBPC} R package. Bioinformatics 2023;39:btac796. 36519837 10.1093/bioinformatics/btac796 PMC9822536

[18] Franko J , Shi Q, Goldman CD, Pockaj BA, Nelson GD, Goldberg RM, . Treatment of colorectal peritoneal carcinomatosis with systemic chemotherapy: a pooled analysis of north central cancer treatment group phase III trials N9741 and N9841. J Clin Oncol 2011;30:263–7. 22162570 10.1200/JCO.2011.37.1039 PMC3269953

[19] Ubink I , van Eden WJ, Snaebjornsson P, Kok NFM, van Kuik J, van Grevenstein WMU, . Histopathological and molecular classification of colorectal cancer and corresponding peritoneal metastases. Br J Surg 2018;105:e204–11. 29341165 10.1002/bjs.10788

[20] Lemmens VE , Klaver YL, Verwaal VJ, Rutten HJ, Coebergh JWW, de Hingh IH. Predictors and survival of synchronous peritoneal carcinomatosis of colorectal origin: a population-based study. Int J Cancer 2011;128:2717–25. 20715167 10.1002/ijc.25596

[21] Kermanshahi TR , Magge D, Choudry H, Ramalingam L, Zhu B, Pingpank J, . Mucinous and signet ring cell differentiation affect patterns of metastasis in colorectal carcinoma and influence survival. Int J Surg Pathol 2017;25:108–17. 27571790 10.1177/1066896916664990

[22] Lurvink RJ , Bakkers C, Rijken A, van Erning FN, Nienhuijs SW, Burger JW, . Increase in the incidence of synchronous and metachronous peritoneal metastases in patients with colorectal cancer: a nationwide study. Eur J Surg Oncol 2021;47:1026–33. 33272737 10.1016/j.ejso.2020.11.135

[23] Soutourina J . Transcription regulation by the Mediator complex. Nat Rev Mol Cell Biol 2018;19:262–74. 29209056 10.1038/nrm.2017.115

[24] Siraj AK , Masoodi T, Bu R, Pratheeshkumar P, Al-Sanea N, Ashari LH, . MED12 is recurrently mutated in Middle Eastern colorectal cancer. Gut 2018;67:663–71. 28183795 10.1136/gutjnl-2016-313334 PMC5868237

[25] Huang S , Hölzel M, Knijnenburg T, Schlicker A, Roepman P, McDermott U, . MED12 controls the response to multiple cancer drugs through regulation of TGF-β receptor signaling. Cell 2012;151:937–50. 23178117 10.1016/j.cell.2012.10.035 PMC3672971

[26] Stein MK , Williard FW, Xiu J, Tsao MW, Martin MG, Deschner BW, . Comprehensive tumor profiling reveals unique molecular differences between peritoneal metastases and primary colorectal adenocarcinoma. J Surg Oncol 2020;121:1320–8. 32166754 10.1002/jso.25899 PMC7505122

[27] Klaus A , Birchmeier W. Wnt signalling and its impact on development and cancer. Nat Rev Cancer 2008;8:387–98. 18432252 10.1038/nrc2389

[28] Zhang L , Shay JW. Multiple roles of APC and its therapeutic implications in colorectal cancer. J Natl Cancer Inst 2017;109:djw332. 28423402 10.1093/jnci/djw332 PMC5963831

[29] Hallam S , Stockton J, Bryer C, Whalley C, Pestinger V, Youssef H, . The transition from primary colorectal cancer to isolated peritoneal malignancy is associated with an increased tumour mutational burden. Sci Rep 2020;10:18900. 33144643 10.1038/s41598-020-75844-6 PMC7641117

[30] El Andaloussi A , Al-Hendy A, Ismail N, Boyer TG, Halder SK. Introduction of somatic mutation in MED12 induces Wnt4/β-catenin and disrupts autophagy in human uterine myometrial cell. Reprod Sci 2020;27:823–32. 32046450 10.1007/s43032-019-00084-7 PMC7539814

[31] Kim S , Xu X, Hecht A, Boyer TG. Mediator is a transducer of Wnt/beta-catenin signaling. J Biol Chem 2006;281:14066–75. 16565090 10.1074/jbc.M602696200

[32] Kawazoe A , Shitara K, Fukuoka S, Kuboki Y, Bando H, Okamoto W, . A retrospective observational study of clinicopathological features of KRAS, NRAS, BRAF and PIK3CA mutations in Japanese patients with metastatic colorectal cancer. BMC Cancer 2015;15:258. 25886136 10.1186/s12885-015-1276-z PMC4393591

[33] Tonello M , Baratti D, Sammartino P, Di Giorgio A, Robella M, Sassaroli C, . Microsatellite and RAS/RAF mutational status as prognostic factors in colorectal peritoneal metastases treated with cytoreductive surgery and Hyperthermic Intraperitoneal Chemotherapy (HIPEC). Ann Surg Oncol 2022;29:3405–17. 34783946 10.1245/s10434-021-11045-3

[34] Diez-Alonso M , Mendoza-Moreno F, Gomez-Sanz R, Matias-Garcia B, Ovejero-Merino E, Molina R, . Prognostic value of KRAS gene mutation on survival of patients with peritoneal metastases of colorectal adenocarcinoma. Int J Surg Oncol 2021;2021:3946875. 34557315 10.1155/2021/3946875 PMC8455216

