# Supplementary figures and images for: Genomic Characterization and Clinical Outcomes of Patients with Peritoneal Metastases from the AACR GENIE Biopharma Collaborative Colorectal Cancer Registry
Source: Cancer Res Commun. 2024 Feb 20;4(2):475–86. doi: 10.1158/2767-9764.CRC-23-0409 (PMC10876516; doi:10.1158/2767-9764.CRC-23-0409)

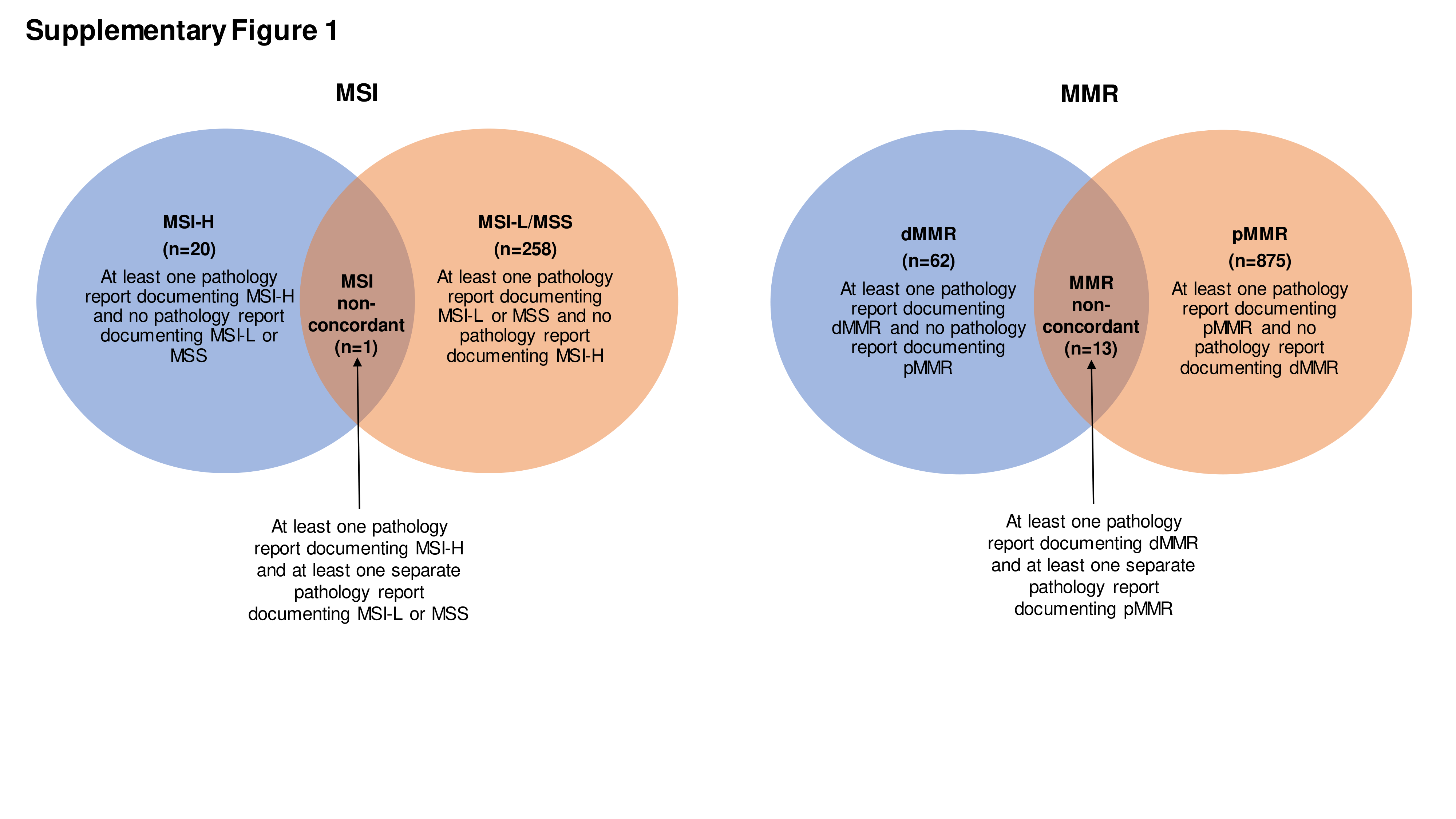

Supplement: Supplementary Figure 1 — Definitions of patient-level MSI and MMR statuses [file crc-23-0409-s01.png]
