# Supplementary material for: Genomic Characterization and Clinical Outcomes of Patients with Peritoneal Metastases from the AACR GENIE Biopharma Collaborative Colorectal Cancer Registry
Source: Cancer Res Commun. 2024 Feb 20;4(2):475–86. doi: 10.1158/2767-9764.CRC-23-0409 (PMC10876516; doi:10.1158/2767-9764.CRC-23-0409)
Supplement: Supplementary Tables — 1-9 [file crc-23-0409-s04.pdf]

Center	Type of Panel	Genes	Depth of coverage	Alteration types	Coverage	Celullarity
DFCI	Custom, hybridation-based capture panel (Oncopanel)	V2 326 genes<br>V3 exonic regions of 447 genes, 191 intronic regions of 60 genes	350x	SNV<br>Small indels<br>CNA (gene level)<br>SVA	Coding exons and Introns	At least 20%
MSKCC	Custom, hybridation-based capture panel (MSK-IMPACT)	V1 341 genes<br><br>V2 410 genes<br><br>V3 468 genes	750X	SNV<br>Small indels<br>CNA (gene and intragenic level)<br>SVA	Coding exons, introns and promoter	At least 10%
VICC	Illumina hybridization-based capture panel (Foundation Medicine)<br><br>Hotspot Amplicon panels (VICC-01-solidtumor)	T5a Panel 326 genes<br><br>T7 Panel 434 genes<br><br>34 genes	750-1000X<br><br>1000X	SNV<br>Small indels<br>CNA (gene level)<br>SVA<br><br>SNV and small indels	Coding exons and introns<br><br>Only Hotspots	At least 20%

Supplementary Table 1. Main genomic analysis performed in GENIE project in the 3 participating centers included in the Colorectal Cancer Cohort. *DCFI: Dana Farber Cancer Center; MSK: Memorial Sloan Kettering Cancer Center; VICC: Vanderbilt-Ingram Cancer Center. SNV: Single Nucleotid Variants; CNA: Copy number alterations; SVA: Structural Variants*

	Stage I-III with Distant Metastasis **(N=581)**		Stage IV **(N = 700)**	
**Characteristic**	**No Peritoneal Metastasis**, N = 486[1]	**Peritoneal Metastasis**, N = 95[1]	**No Peritoneal Metastasis**, N = 551[1]	**Peritoneal Metastasis**, N = 149[1]
**Age at diagnosis**	56 (48, 66)	52 (48, 64)	53 (45, 63)	53 (44, 63)
**Sex**				
**Female**	193 (40%)	55 (58%)	239 (43%)	84 (56%)
**Male**	293 (60%)	40 (42%)	312 (57%)	65 (44%)
**Race/ethnicity**				
**Non-Hispanic White**	386 (79%)	73 (77%)	415 (75%)	108 (72%)
**Non-Hispanic Black**	33 (6.8%)	8 (8.4%)	42 (7.6%)	8 (5.4%)
**AAAPI (Asian, Asian American, and Pacific Islander)**	22 (4.5%)	7 (7.4%)	33 (6.0%)	9 (6.0%)
**Unknown race**	23 (4.7%)	2 (2.1%)	24 (4.4%)	10 (6.7%)
**Hispanic/Latinx**	15 (3.1%)	4 (4.2%)	25 (4.5%)	6 (4.0%)
**Other**	7 (1.4%)	1 (1.1%)	12 (2.2%)	8 (5.4%)
**Primary tumor**				
**Location of primary tumor**				
**Left colon**	124 (27%)	30 (33%)	185 (35%)	49 (36%)
**Rectal**	205 (44%)	21 (23%)	184 (35%)	21 (16%)
**Right colon**	137 (29%)	41 (45%)	155 (30%)	65 (48%)
**Unknown**	20	3	27	14
**Side of primary tumor**				
**Left**	329 (71%)	51 (55%)	369 (70%)	70 (52%)
**Right**	137 (29%)	41 (45%)	155 (30%)	65 (48%)
**Unknown**	20	3	27	14
**Histological grade**				
**I/II**	319 (75%)	53 (64%)	371 (79%)	69 (58%)
**III/IV**	106 (25%)	30 (36%)	100 (21%)	49 (42%)
**Unknown**	61	12	80	31
**Histology**				
**Adenocarcinoma**	378 (91%)	63 (76%)	453 (96%)	102 (81%)
**Mucinous adenocarcinoma**	24 (5.8%)	16 (19%)	10 (2.1%)	17 (13%)
**Other histologies/mixed tumor**	8 (1.9%)	0 (0%)	8 (1.7%)	2 (1.6%)

**Signet ring cell carcinoma**	4 (1.0%)	4 (4.8%)	3 (0.6%)	5 (4.0%)
**Unknown**	72	12	77	23
**MSI status**				
**MSI-H**	11 (2%)	2 (2%)	4 (3.9%)	3 (7.1%)
**MSI-L/MSS**	105 (22%)	16 (17%)	98 (96%)	39 (93%)
**MSI non-concordant**	0 (0%)	1 (1%)	0 (0%)	0 (0%)
**Unknown MSI status**	370 (76%)	76 (80%)	449	107
**MMR status**				
**dMMR**	39 (8%)	5 (6.3%)	8 (2.0%)	10 (9.0%)
**Indeterminate**	5 (1%)	0 (0%)	1 (0.2%)	2 (1.8%)
**pMMR**	316 (65%)	72 (90%)	388	99
**MMR non-concordant**	6 (1%)	3 (3.8%)	4 (1.0%)	0 (0%)
**Unknown MMR status**	120 (25%)	15	150	38
**NGS report after death/last follow-up**	27 (5.6%)	5 (5.3%)	36 (6.5%)	9 (6.0%)
**Sites of initial metastases (at the time of diagnosis with either stage IV disease or presentation with metastases following stage I-III)**				
Liver metastases	170 (36%)	9 (9.5%)	490 (92%)	54 (36%)
Unknown	8	0	18	0
Lung metastases	124 (26%)	5 (5.3%)	113 (21%)	24 (16%)
Unknown	8	0	18	0
Brain metastases	2 (0.4%)	0 (0%)	6 (1.1%)	0 (0%)
Unknown	8	0	18	0
Bone metastases	10 (2.1%)	0 (0%)	19 (3.6%)	6 (4.0%)
Unknown	8	0	18	0

[1]n (%); Median (IQR)

Supplementary Table 2: Main clinical, pathological, and molecular characteristics of the population included in the study according to stage I-III or IV at diagnosis: Association between those variables and presence of peritoneal metastases (PM) has been also analyzed (p-value). *dMMR: Deficient mismatch*

repair system; IQR: interquartile range; pMMR: proficient mismatch repair system. Sd: Standard deviation; min: minimum; max: maximum.

**Characteristic**	Overall, **N = 1,281**[1]	Stage I-III with Distant Metastasis, **N = 581**[1]	Stage IV, **N = 700**[1]
**Sample type**			
**Local recurrence**	12 (0.9%)	10 (1.7%)	2 (0.3%)
**Lymph node metastasis**	2 (0.2%)	2 (0.3%)	0 (0%)
**Metastasis site unspecified**	469 (37%)	213 (37%)	256 (37%)
**Unknown**	6 (0.5%)	4 (0.7%)	2 (0.3%)
**Primary tumor**	792 (62%)	352 (61%)	440 (63%)
**Sequencing Assay ID**			
**DFCI-ONCOPANEL-2**	241 (19%)	126 (22%)	115 (16%)
**DFCI-ONCOPANEL-3**	224 (17%)	120 (21%)	104 (15%)
**MSK-IMPACT341**	7 (0.5%)	2 (0.3%)	5 (0.7%)
**MSK-IMPACT410**	290 (23%)	123 (21%)	167 (24%)
**MSK-IMPACT468**	300 (23%)	124 (21%)	176 (25%)
**VICC-01-SOLIDTUMOR**	52 (4.1%)	20 (3.4%)	32 (4.6%)
**VICC-01-T5A**	1 (<0.1%)	0 (0%)	1 (0.1%)
**VICC-01-T7**	166 (13%)	66 (11%)	100 (14%)

[1]n (%)

Supplementary Table 3: Samples analyzed for next generation sequencing (NGS), origin of the sample and panel NGS used.

Characteristic	Overall, N = 1,204[1]	No Peritoneal Metastasis, N = 974[1]	Peritoneal Metastasis, N = 230[1]
Metastatic cohort			
Stage I-III with Distant Metastasis	549 (46%)	459 (47%)	90 (39%)
Stage IV	655 (54%)	515 (53%)	140 (61%)
Age at diagnosis	54 (46, 64)	54 (47, 64)	52 (45, 63)
Sex			
Female	538 (45%)	407 (42%)	131 (57%)
Male	666 (55%)	567 (58%)	99 (43%)
Race/ethnicity			
Non-Hispanic White	972 (77%)	757 (78%)	172 (75%)
Non-Hispanic Black	86 (7.1%)	70 (7.2%)	16 (7.0%)
AAAPI (Asian, Asian American, and Pacific Islander)	67 (5.6%)	51 (5.2%)	16 (7.0%)
Unknown race	50 (4.2%)	42 (4.3%)	8 (3.5%)
Hispanic/Latinx	47 (3.9%)	37 (3.8%)	10 (4.3%)
Other	25 (2.1%)	17 (1.7%)	8 (3.5%)
**Primary tumor**			
Location of primary tumor			
Left colon	368 (32%)	294 (32%)	74 (34%)
Rectal	407 (36%)	366 (39%)	41 (19%)
Right colon	369 (32%)	269 (29%)	100 (47%)
Unknown	60	45	15
Side of primary tumor			
Left	775 (68%)	660 (71%)	115 (53%)
Right	369 (32%)	269 (29%)	100 (47%)
Unknown	60	45	15
Histological grade			
I/II	769 (74%)	653 (77%)	116 (61%)
III/IV	265 (26%)	192 (23%)	73 (39%)
Unknown	170	129	41
Histology			
Adenocarcinoma	966 (91%)	803 (94%)	163 (80%)
Mucinous adenocarcinoma	64 (6.0%)	33 (3.8%)	31 (15%)
Other histologies/mixed tumor	18 (1.7%)	16 (1.9%)	2 (1.0%)
Signet ring cell carcinoma	15 (1.4%)	6 (0.7%)	9 (4.4%)
Unknown	141	116	25
MSI status			
MSI-H	16 (6.2%)	13 (6.4%)	3 (5.6%)
MSI-L/MSS	240 (93%)	190 (94%)	50 (93%)
MSI non-concordant	1 (<1%)	0 (0%)	1 (2%)
Unknown	947	771	176
MMR status			
dMMR	58 (6.4%)	45 (6.2%)	13 (7.1%)
pMMR	824 (91%)	660 (92%)	164 (90%)
Indeterminate	8 (0.9%)	6 (0.8%)	2 (1.1%)
MMR non-concordant	12 (1.3%)	9 (1.3%)	3 (1.6%)
Unknown	302	254	48
NGS report after death/last follow-up	0 (0%)	0 (0%)	0 (0%)
**Sites of initial metastases (at the time of diagnosis with either stage IV disease or presentation with metastases following stage I-III)**			
Liver metastases	684 (58%)	624 (66%)	60 (26%)
Unknown	25	25	0
Lung metastases	249 (21%)	220 (23%)	29 (13%)
Unknown	25	25	0
Brain metastases	8 (0.7%)	8 (0.8%)	0 (0%)
Unknown	25	25	0
Bone metastases	31 (2.6%)	25 (2.6%)	6 (2.6%)
Unknown	25	25	0

[1]n (%); Median (IQR)

Supplementary Table 4: Main clinical, pathological, and molecular characteristics of the population included in the OS cohort: Association between those variables and presence of PM has been also analyzed (p-value). *dMMR: Deficient mismatch repair system; pMMR: proficient mismatch repair system.*

Characteristic	N	N event	Median overall survival (months)	p-value[1]
KRAS status by presence of PM				<0.001
KRAS mt; No PM	423	246	27.24 (21.74, 33.32)	
KRAS mt; PM	116	72	23.72 (17.60, 31.71)	
KRAS wt; No PM	550	273	40.53 (34.21, 45.33)	
KRAS wt; PM	114	65	28.09 (24.47, 38.49)	
BRAF status by presence of PM				0.035
BRAF mt; No PM	92	51	20.92 (16.58, 32.17)	
BRAF mt; PM	26	17	21.97 (12.93, 49.97)	
BRAF wt; No PM	881	468	34.21 (31.32, 39.01)	
BRAF wt; PM	204	120	26.78 (22.66, 31.81)	
NRAS status by presence of PM				0.035
NRAS mt; No PM	51	29	30.39 (25.99, 48.42)	
NRAS mt; PM	8	5	28.09 (14.44, —)	
NRAS wt; No PM	922	490	33.29 (30.43, 37.96)	
NRAS wt; PM	222	132	26.78 (22.01, 31.28)	
RAS/BRAF status by presence of PM				<0.001
RAS/BRAF mt; No PM	547	319	26.71 (23.26, 31.51)	
RAS/BRAF mt; PM	146	91	23.13 (18.75, 30.30)	
RAS/BRAF wt; No PM	426	200	45.03 (40.23, 54.28)	
RAS/BRAF wt; PM	84	46	29.14 (26.58, 42.43)	
PIK3CA status by presence of PM				0.013
PIK3CA mt; No PM	203	96	36.32 (28.49, 49.64)	
PIK3CA mt; PM	48	27	29.41 (24.84, 43.98)	
PIK3CA wt; No PM	770	423	32.34 (29.74, 37.73)	
PIK3CA wt; PM	182	110	24.80 (19.57, 31.12)	
APC status by presence of PM				0.001
APC mt; No PM	740	378	36.35 (31.61, 40.69)	
APC mt; PM	141	81	29.41 (24.47, 37.37)	
APC wt; No PM	190	113	25.72 (20.59, 33.32)	
APC wt; PM	82	51	23.72 (17.60, 31.12)	

Unknown	50	33		
TP53 status by presence of PM				0.025
TP53 mt; No PM	719	378	34.38 (31.45, 39.24)	
TP53 mt; PM	156	94	24.47 (19.77, 31.12)	
TP53 wt; No PM	254	141	29.74 (23.26, 38.98)	
TP53 wt; PM	74	43	29.41 (24.54, 40.56)	

[1]Log-rank p-value

Supplementary Table 5: Overall survival according to *KRAS*, *NRAS* and *BRAF* status and the presence of peritoneal metastases. *PM: Peritoneal metastases; non-PM: No Peritoneal metastases. Note: estimations of genomic alterations presented are not annotated according to OncoKB.*

Drug Regimen	N = 473[1]
FOLFOX	218 (46.3%)
FOLFOX + Bevacizumab	94 (19.9%)
FOLFIRI + Bevacizumab	78 (16.5%)
FOLFIRI	46 (9.7%)
FOLFOXIRI	11 (2.3%)
XELOX	8 (1.7%)
FOLFOXIRI + Bevacizumab	6 (1.3%)
XELOX + Bevacizumab	5 (1%)
FOLFIRI + Panitumumab	3 (0.6%)
FOLFOX + Panitumumab	3 (0.6%)
FOLFIRI + Cetuximab	1 (0.2%)

[1]n (%)

Supplementary Table 6: Systemic therapy offered in first line therapy in combination for mCRC in the GENIE BPC cohort. *FOLFOX: 5-fluouracile + leucovorin + oxaliplatin; FOLFIRI: 5-fluouracile + leucovorin + oxaliplatin; FOLFOXIRI: 5-fluouracile + leucovorin + oxaliplatin + irinotecan; XELOX: Capecitabine + Oxaliplatin.*

Characteristic	N	N event	Median PFS (months)
**PM vs. no PM**			
PFS-I			
No PM	344	171	9.572 (8.191, 11.45)
PM	93	49	8.882 (7.829, 13.62)
PFS-M			
No PM	365	138	13.32 (11.38, 16.58)
PM	100	41	11.68 (8.289, 18.91)
PFS-I-or-M			
No PM	337	180	8.454 (7.368, 9.901)
PM	92	53	7.928 (5.526, 10.23)
**PM only vs. PM and other metastasis vs. no PM**			
PFS-I			
No PM	372	129	9.572 (8.191, 11.45)
PM and others	47	17	7.829 (2.829, 9.309)
PM only	54	20	10.53 (8.421, 31.41)
PFS-M			
No PM	344	171	13.32 (11.38, 16.58)
PM and others	45	26	6.678 (1.053, —)
PM only	48	23	13.82 (10.59, 34.24)
PFS-I-or-M			
No PM	377	180	8.454 (7.368, 9.901)
PM and others	44	28	5.362 (1.612, 8.717)
PM only	48	25	10.23 (7.434, 16.35)

Supplementary Table 7: PFS based on different approaches in patients with peritoneal metastases (PM) or without PM (non-PM). *PFS-I: Progression Free Survival based on imaging. PFS-M: Progression Free Survival based on Medical Reports; PFS-I-or-M: Progression Free Survival based on the earlier of PFS-I and PFS-M, whichever occurred first.*

Characteristic	N	N event	Median PFS (months)	p-value[1]
KRAS status by presence of PM				0.281
KRAS mt; No PM	169	63	13.26 (10.46, 20.03)	
KRAS mt; PM	55	18	13.82 (8.914, —)	
KRAS wt; No PM	203	66	15.30 (13.62, 22.43)	
KRAS wt; PM	46	19	18.91 (9.507, —)	
BRAF status by presence of PM				0.440
BRAF mt; No PM	31	10	22.57 (10.16, —)	
BRAF mt; PM	11	8	7.993 (4.408, —)	
BRAF wt; No PM	341	119	14.90 (13.26, 17.43)	
BRAF wt; PM	90	29	13.88 (11.38, 34.24)	
RAS/BRAF status by presence of PM				0.152
RAS/BRAF mt; No PM	217	78	13.98 (11.51, 20.03)	
RAS/BRAF mt; PM	67	25	10.59 (8.289, 23.13)	
RAS/BRAF wt; No PM	155	51	15.43 (13.62, 22.43)	
RAS/BRAF wt; PM	34	12	18.91 (11.38, —)	
PIK3CA status by presence of PM				0.554
PIK3CA mt; No PM	82	27	14.47 (11.84, —)	
PIK3CA mt; PM	16	5	— (9.507, —)	
PIK3CA wt; No PM	290	102	15.16 (13.22, 19.28)	
PIK3CA wt; PM	85	32	13.82 (10.59, 26.51)	
APC status by presence of PM				0.199
APC mt; No PM	287	99	15.30 (13.45, 20.72)	
APC mt; PM	56	18	21.71 (9.507, —)	
APC wt; No PM	64	24	10.10 (9.539, 19.28)	
APC wt; PM	43	18	11.68 (8.421, —)	
Unknown	23	7		
TP53 status by presence of PM				0.588
TP53 mt; No PM	276	96	14.24 (13.22, 17.43)	
TP53 mt; PM	69	26	13.88 (9.276, 31.58)	
TP53 wt; No PM	96	33	16.58 (11.84, —)	
TP53 wt; PM	32	11	13.82 (11.38, —)	

[1]Log-rank p-value

Supplementary Table 8: Progression-free survival according to *KRAS*, *NRAS*, *BRAF, PI3KCA, APC and TP53* status and the presence of Peritoneal Metastases. *PM: Peritoneal metastases; non-PM: No Peritoneal metastases; NA: Not available*

Univariable Cox Proportional Hazards Models					
**Characteristic**	**N**	**Event N**	**HR[1]**	**95% CI[1]**	**p-value**
Presence of peritoneal metastasis					0.5
No peritoneal metastasis	372	129	—	—	
Peritoneal metastasis	101	37	1.15	0.80, 1.65	
Presence of peritoneal metastasis (peritoneal only vs. peritoneal and other sites of distant metastasis vs. no peritoneal metastasis)					0.4
No peritoneal metastasis	372	129	—	—	
Peritoneal metastasis and other sites of distant metastasis	47	17	1.41	0.85, 2.34	
Peritoneal metastasis only	54	20	0.99	0.62, 1.59	
Metastatic cohort					0.3
Stage I-III with Distant Metastases	173	69	—	—	
Stage IV	300	97	1.18	0.86, 1.61	
Location of primary tumor					0.7
Left colon	145	54	—	—	
Rectum	149	45	0.83	0.56, 1.24	
Right colon	157	59	0.93	0.64, 1.35	
Side of primary tumor					>0.9
Left	294	99	—	—	
Right	157	59	1.02	0.74, 1.40	
MSI status					>0.9
MSI-L/MSS	85	27	—	—	
MSI-H	5	2	0.96	0.22, 4.18	
MMR status					0.3
pMMR	350	121	—	—	
dMMR	14	5	0.63	0.26, 1.53	
Any *KRAS/BRAF/NRAS* alteration	284	103	1.32	0.96, 1.80	0.087
APC alteration	343	117	0.69	0.48, 0.98	**0.040**
TP53 alteration	345	122	1.20	0.85, 1.69	0.3
PIK3CA alteration	98	32	0.87	0.59, 1.28	0.5
Liver metastases at advanced diagnosis	282	98	1.37	1.00, 1.88	**0.049**
Lung metastases at advanced diagnosis	112	37	1.24	0.86, 1.80	0.2
Multivariable Cox Proportional Hazards Model					
**Characteristic**			**HR[1]**	**95% CI[1]**	**p-value**
Time (months) from advanced diagnosis to NGS report	442	157	0.99	0.94, 1.04	0.7
APC alteration	267	95	0.61	0.42, 0.89	**0.010**
Liver metastases at advanced diagnosis	338	117	1.70	1.22, 2.38	**0.002**

[1]HR = Hazard Ratio, CI = Confidence Interval

**Note: 2 patients in the PFS study cohort with MMR non-concordant status were omitted from the univariable analysis.*

Supplementary Table 9. Univariable and Multivariable Cox proportional hazards models for progression-free survival. *PM: Peritoneal metastasis; non-PM: No Peritoneal metastasis; dMMR: Deficient mismatch repair system; MSI-H: MSI-high; MSI-L/MSS: MSI-low/microsatellite stable; pMMR: proficient mismatch repair system.*
